# Genome-Wide Characterization and Expression Profiling of the *CCR* Gene Family Associated with Stem Strength in Upland Cotton (*Gossypium hirsutum* L.)

**DOI:** 10.3390/life16050861

**Published:** 2026-05-21

**Authors:** Cong-Hua Feng, Dan Li, Suen Liu, Linlin Liu, Cunpeng Zhao, Kaihui Wang, Di Liu, Haina Zhang, Jina Chi, Yuyuan Qian, Xinlong Gao, Yi Liu, Junyi Geng, Baosheng Guo

**Affiliations:** Institute of Cotton, Hebei Academy of Agriculture and Forestry Sciences, Key Laboratory of Cotton Biology and Genetic Breeding in Huanghuaihai Semiarid Area, Ministry of Agriculture and Rural Affairs, Hebei Key Laboratory of Cotton Bio-Breeding and Cultivation Physiology, Shijiazhuang 050051, China; fch19942024@163.com (C.-H.F.); lidan@haafs.org (D.L.); liusuen@haafs.org (S.L.); spp2016@126.com (L.L.); zhaocunpeng@haafs.org (C.Z.); wangkaihui@haafs.org (K.W.); liudijoy@126.com (D.L.); zhanghaina@haafs.org (H.Z.); chijina2021@163.com (J.C.); qyyuan8@163.com (Y.Q.); gaoxl4work@gmail.com (X.G.); liuyi1105@126.com (Y.L.)

**Keywords:** *Gossypium hirsutum*, cinnamoyl-CoA reductase (CCR), lignin biosynthesis, stem strength, lodging resistance, abiotic stress, gene expression

## Abstract

In this study, we performed the first genome-wide identification and characterization of the cinnamoyl-CoA reductase (*CCR*) gene family in upland cotton (*Gossypium hirsutum*), focusing on its potential association with stem strength. We identified 76 *GhCCR* genes and classified them into four subfamilies. We then analyzed their evolutionary relationships, conserved domains, synteny, promoter cis-elements, and expression patterns. All GhCCR proteins possess the NADB_Rossmann superfamily domain, and family expansion appears to have been driven mainly by segmental and tandem duplications. A small number of *GhCCR* genes showed relatively high expression in leaf, pistil, and torus tissues, while genes such as *GhCCR3/9/10* exhibited elevated transcript levels under abiotic stress conditions. RT-qPCR results indicated that three candidate *GhCCR* genes (*GhCCR25*, *GhCCR52* and *GhCCR64*) were significantly more highly expressed in multiple tissues of the stiff-stem line *JY-25* than in the soft-stem line *JR-15*. Together, these findings suggest that *GhCCR* genes may contribute to the regulation of growth, development, and stress adaptation in *G. hirsutum*. However, direct biochemical or genetic validation is required to confirm their functional roles in lignin biosynthesis and stem rigidity.

## 1. Introduction

Cotton, the world’s most crucial natural textile raw material, is integral to the global agricultural and trade landscape due to its diverse applications in fiber, food, and feed [1,2]. However, its productivity remains highly vulnerable to abiotic stresses such as cold, drought, and salinity, which can severely reduce yields [3,4,5]. Furthermore, lodging—stem bending or breaking under strong winds—poses a significant threat to both yield and quality, especially during harvest. Lodging not only reduces yield and fiber quality but also severely impairs mechanical harvesting efficiency, as bent or broken stalks cannot be picked cleanly, leading to increased harvest losses and higher production costs [6,7]. Stem strength in cotton is determined by a complex interplay of genetic, physiological, epigenetic, and environmental factors. Key mechanical properties, including rigidity and strength, are strongly correlated with lignin content and are further modulated by the composition of lignin monomers and their interactions with other cell wall components [8,9]. With climate change expected to exacerbate these environmental challenges, developing resilient cotton varieties has become an urgent priority for ensuring the sustainability of global cotton production [10].

Plant cell walls constitute a highly dynamic and precisely regulated extracellular matrix capable of real-time perception of morphogenetic signals and integration of diverse stress-induced environmental changes [11,12]. The directed deposition of lignin within secondary cell walls is the primary mechanism underpinning cell wall thickening and enhanced mechanical strength, serving as a core physiological module for plant adaptation to terrestrial environments and multiple stresses by synergistically enhancing cell wall integrity, regulating water and solute dynamics, and indirectly stabilizing membrane structures [13,14,15]. Stress signals such as abscisic acid (ABA) and reactive oxygen species (ROS) rapidly activate the phenylpropanoid pathway, elevating the activity of key enzymes including phenylalanine ammonia-lyase (*PAL*), 4-coumarate: coenzyme A ligase (*4CL*), cinnamoyl-CoA reductase (*CCR*), and cinnamyl alcohol dehydrogenase (*CAD*), leading to extensive polymerization of lignin monomers within xylem vessels, fibers, and root endodermis [16,17,18,19]. Lignin synthesis genes play crucial roles across species: in *Oryza sativa*, *Os4CL4* enhances aluminum tolerance by modifying cell walls to reduce aluminum binding, while *OsPAL8* overexpression boosts resistance via salicylic acid and lignin synthesis pathways under regulation by *OsMYB30* [17]; in *Triticum aestivum*, *TaCAD12* overexpression upregulates defense and lignin synthesis genes to combat Fusarium wilt [20]; and in *Solanum tuberosum*, *StPAL1* expression is significantly upregulated by drought, high temperature, and methyl jasmonate (MeJA), indicating its role in abiotic stress defense [21].

The *CCR* gene family encodes the first key enzyme in the lignin-specific biosynthesis pathway. Unlike *PAL* and *4CL*, which are involved in multiple phenylpropanoid pathways, *CCR* acts as the first dedicated enzyme for monolignol biosynthesis and is considered a key rate-limiting step specifically for lignin formation [13,18]. Gene copy numbers vary across species, with *Triticum aestivum* possessing 20, *Capsicum* spp. 38, and *Dalbergia odorifera* 24 [22,23,24]. The *CCR* gene families of *Dalbergia odorifera*, pepper (*Capsicum chinense*) and wheat (*Triticum aestivum*) all exhibit sequence conservation, with their core members being involved in lignin biosynthesis. Their promoter regions all contain cis-acting elements associated with stress, light, hormone and growth/development responses, and the genes show tissue-specific expression patterns with inducible expression in response to external stimuli. However, the *CCR* gene families of these three species vary considerably in terms of their size, classification and chromosomal distribution. These *CCR* genes significantly influence plant growth, development, and stress resistance. For example, in *O. sativa*, *OsCCR10* is directly activated by the transcription factor *OsNAC5*, enhancing drought tolerance during vegetative growth by regulating lignin accumulation [25]. Concurrently, the expression of *OsCCR17* and *OsCCR21* is induced by both biotic and abiotic stresses, indicating their defensive roles [26]. In *B. napus*, overexpression of *BnaCCR2.b* increased stem lignin content by 2.28–2.76%, thereby boosting resistance to *Sclerotinia sclerotiorum* [27]. In the economically important tree species *Morus alba*, downregulation of *MaCCR1* altered lignin content and stunted growth [28]. Conversely, reduced expression of the maize *CCR1* gene did not affect overall growth but led to a slight decrease in lignin content and significant structural alterations [29]. These findings collectively indicate a potential pleiotropic role of *CCR* genes in coordinating plant development and stress adaptation, which may underpin their strategic value as candidate targets for molecular breeding [18].

Based on the long-completed whole-genome sequencing of *G. hirsutum*, this study systematically identified the *GhCCR* gene family, addressing a current research gap. We characterized its phylogenetic relationships, conserved domains, and gene structures. Transcriptomic analysis revealed tissue-specific variations in *GhCCR* transcript abundance. Expression profiling under three abiotic stresses (cold, NaCl, and PEG-simulated drought) further identified key *GhCCR* candidates implicated in growth regulation and stress resistance. RT-qPCR results showed that expression levels of three *GhCCR* genes (*GhCCR25*, *GhCCR52*, and *GhCCR64*) were significantly higher in the roots and stems of a stiff-stem line (*JY-25*) than in a soft-stem line (*JR-15*), suggesting their possible role in promoting lignin synthesis. These findings offer preliminary insights into the *GhCCR* gene family’s function in regulating stem strength and provide a theoretical foundation for future mechanistic research in cotton.

## 2. Results

### 2.1. Identification and Phylogenetic Analysis of the GhCCR Gene Family

**Figure 1:**
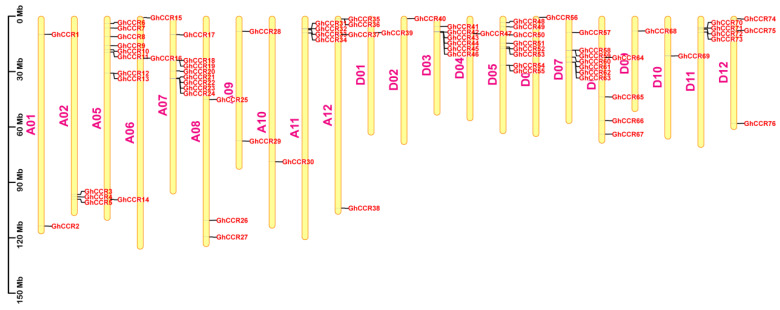
Chromosomal distribution of *GhCCR* genes in *G. hirsutum*. The scale bar on the left represents a chromosomal length of 30 Mb. Individual chromosomes are depicted as yellow rectangles, with their corresponding codes labeled alongside.

To identify *GhCCR* genes, we performed BLAST (TBtools_windows-x64_2_376) searches using known CCR protein sequences from *A. thaliana* as queries against the *G. hirsutum* genome. The search was conducted with an E-value threshold of 1 × 10^−10^ and a minimum sequence identity of 50%. Candidate hits were further validated by confirming the presence of the conserved NADB_Rossmann domain and the FR_SDR_e domain using NCBI CDD and Pfam databases. A total of 76 *GhCCR* genes were identified and systematically named *GhCCR1* to *GhCCR76* based on their chromosomal order (Table S1). These genes are distributed across 22 chromosomes (Figure 1). Chromosome A05 contains the most members (nine genes), while chromosomes A10, D01, D02, D06, D09, and D10 each harbor only a single *GhCCR* gene. Nine tandem repeat clusters were identified, with the largest cluster containing four consecutive genes (*GhCCR42–GhCCR45*) (Table S2). Analysis of physicochemical properties shows that GhCCR proteins vary in length from 97 to 596 amino acids, with molecular weights exceeding 10 kDa (Table S1). Their theoretical isoelectric points (pI) range from 5.02 to 9.33, with 21 genes (28%) encoding basic proteins. Only eight members (~11%) are predicted to be unstable (instability index > 40). In terms of hydrophobicity, 63% of GhCCR proteins are hydrophilic and 37% are hydrophobic. Subcellular localization predictions (Plant-mPLoc) indicate that the majority of GhCCR proteins are targeted to the Golgi apparatus. Eight genes (*GhCCR3*, *5*, *12*, *36*, *42*, *43*, *44*, *61*) are predicted to localize to the chloroplast, cytoplasm, or nucleus.

**Figure 2:**
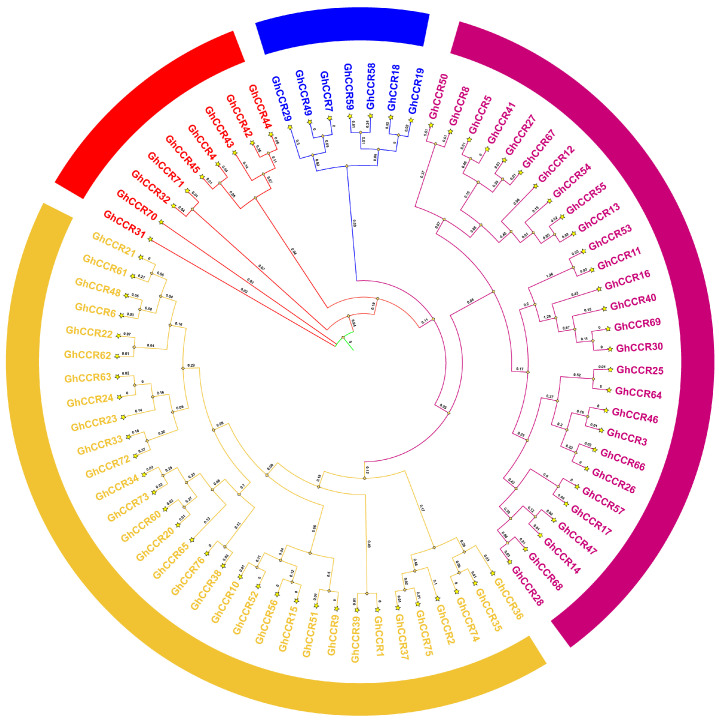
Phylogenetic and comparative analysis of the *CCR* gene family. Phylogenetic tree of GhCCR proteins in *G. hirsutum*. The proteins are classified into four distinct subfamilies, indicated by different colored branches.

Phylogenetic analysis was performed using the Neighbor-Joining (NJ) method in MEGA7.0 with 5000 bootstrap replicates. The NJ method was chosen because it is computationally efficient for large datasets and produced consistent topologies when compared with maximum likelihood (ML) analysis. This analysis classified the 76 GhCCR proteins into four distinct subfamilies: I (7 members), II (28 members), III (32 members), and IV (9 members) (Figure 2).

### 2.2. Analysis of Conserved Motifs, Protein Domains, and Gene Structure of GhCCR Genes

**Figure 3:**
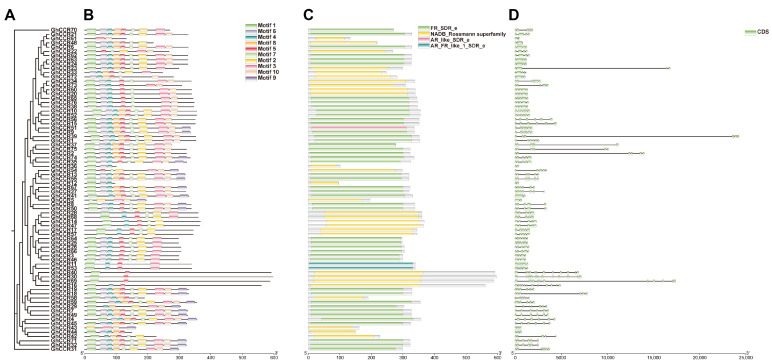
Comprehensive analysis of *GhCCR* genes in *G. hirsutum*: (**A**) Phylogenetic tree. (**B**) Distribution of conserved protein motifs, with different colors representing distinct motifs. (**C**) Architecture of conserved protein domains, with different colors indicating different domains. (**D**) Gene structure, where green rectangles represent exons and black lines represent introns.

**Figure 4:**
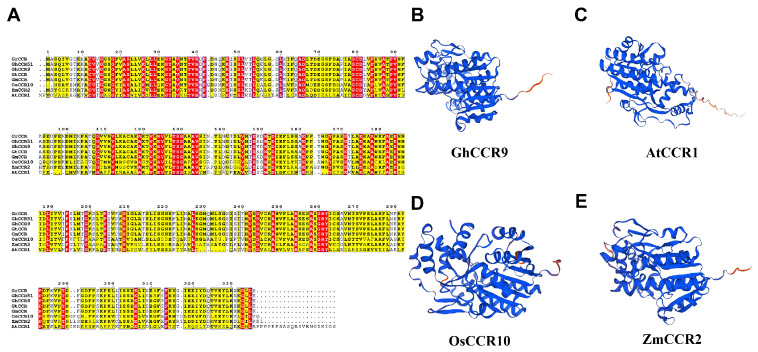
Multiple sequence alignment and structural analysis of *GhCCR9*: (**A**) Red text denotes conserved sequences, with red highlighting indicating identical sequences. The yellow highlighted parts indicate a relatively high sequence similarity, but there are some differences in amino acids. *GhCCR9* (*G. hirsutum*); *GhCCR51* (*G. hirsutum*); *GtCCR* (*Gossypium tomentosum*, *Gotom.A05G181700.1*); *GmCCR* (*Gossypium mustelinum*, *Gomus.A05G179300.1*); *GrCCR* (*Gossypium raimondii*, *Gorai.009G175500.1*); *AtCCR1* (*Arabidopsis thaliana*, *AT1G15950.1*); *OsCCR10* (*Oryza sativa*, *LOC_Os02g09960.1*); *ZmCCR2* (*Zea mays*, *ZmB84.10G220400.1*). (**B**–**E**) Tertiary structures of *CCR* genes from four species. (**B**) *GhCCR9*; (**C**) *AtCCR1*; (**D**) *OsCCR10*; (**E**) *ZmCCR2*.

Analysis of conserved protein motifs revealed that most of the 76 *GhCCR* members contain approximately eight conserved motifs, with Motif 1 and Motif 5 being the most prevalent (Figure 3B, Table S3). Genes clustered on the same phylogenetic branch tend to share highly similar motif compositions, indicating strong sequence conservation within subfamilies. Domain architecture analysis reveals that the majority of members in this gene family harbor the conserved FR_SDR_e domain, with a large proportion additionally carrying the NADB_Rossmann superfamily domain. Multiple sequence alignment revealed that all 76 GhCCR proteins contain the canonical NAD(P)H-binding motif (VTGALFGKT) and the conserved active-site residues characteristic of *CCR* enzymes, indicating that the catalytic core is preserved across the entire family (Figure 3B,C). Gene structure analysis shows that *GhCCR* genes contain 2 to 12 exons and 1 to 11 introns (Figure 3D). Cross-species sequence alignment and protein tertiary structure comparison revealed that CCR proteins are highly conserved (Figure 4A–E and Figure S2). The predicted tertiary structures (Figure 4B–E and Figure S2B–D) show that *GhCCR9*, *GhCCR51*, *AtCCR1*, *OsCCR10*, *ZmCCR2 AtCCR2*, *GhCCR8* and *GhCCR50* all adopt a typical Rossmann fold, characterized by a central β-sheet flanked by α-helices, which is consistent with their conserved NAD(P)H-binding and catalytic functions. The high structural similarity among these distantly related species further supports the functional conservation of *CCR* enzymes in lignin biosynthesis. Ka/Ks analysis indicates that *GhCCRs* are under purifying selection.

### 2.3. Collinearity Analysis of the CCR Gene Family in G. hirsutum

**Figure 5:**
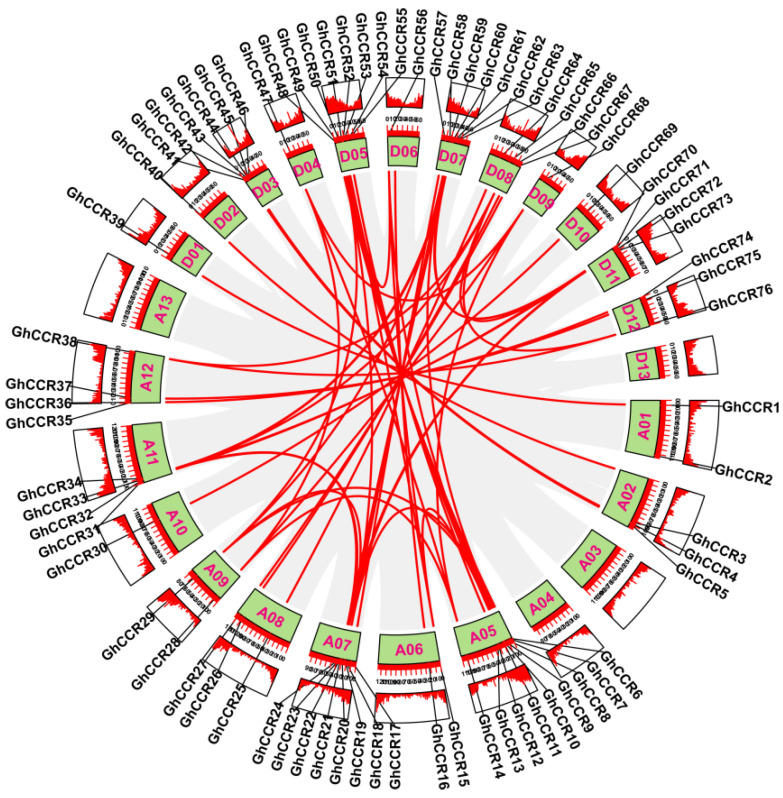
Intra-genomic collinearity analysis of *GhCCR* genes in *G. hirsutum*. The inner ring displays chromosomes as green arcs of proportional lengths. Gene density along each chromosome is represented by the outer heatmap ring. Gray lines in the background depict systemic blocks across the genome, while red lines highlight collinear *GhCCR* gene pairs.

**Figure 6:**
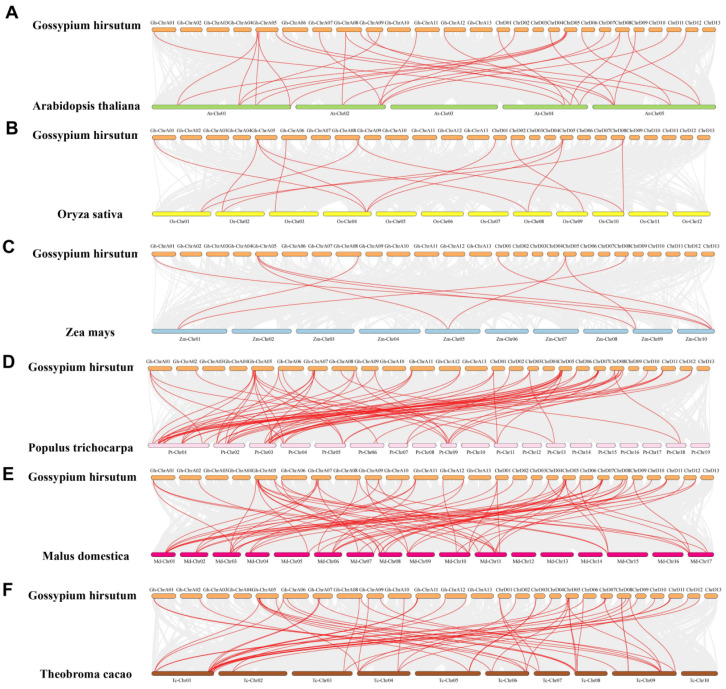
(**A**–**F**) Comparative synteny analysis of *CCR* genes between *G. hirsutum* and six representative plant species. The gray lines in each panel represent systemic blocks between *G. hirsutum* and the compared species (*A. thaliana*, *O. sativa*, *Z. mays*, *P. trichocarpa*, *M. domestica*, and *T. cacao*), while the red lines highlight homologous *CCR* gene pairs.

The *GhCCR* gene family in *G. hirsutum* has expanded through multiple duplication events during evolution. Within the cotton genome, the 76 *GhCCR* genes formed 58 segmentally duplicated gene pairs (Figure 5). Comparative synteny analysis was further performed between *G. hirsutum* and six other plant species: *A. thaliana*, *O. sativa*, *Z. mays*, *P. trichocarpa*, *Malus domestica*, *Theobroma cacao*, *Brassica napus*, *Cucumis sativus* and *Medicago sativa* (Figure 6A–F and Figure S3). This analysis identified numerous homologous gene pairs: 30 with *A. thaliana*, 14 with *O. sativa*, 9 with *Z. mays*, 69 with *P. trichocarpa*, 55 with *M. domestica*, 55 with *T. cacao*, 43 with *B. napus*, 41 with *C. sativus* and 29 with *M. sativa*.

### 2.4. Analysis of Cis-Acting Elements Within the GhCCR Gene Family

**Figure 7:**
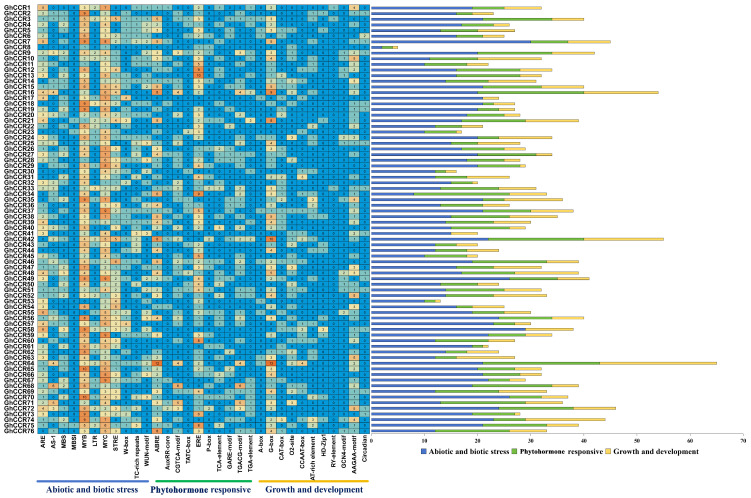
Analysis of cis-acting regulatory elements in GhCCR gene promoters. The heatmap illustrates the abundance of each element type across all GhCCR genes, with color gradient and numbers indicating the count. The colored bar charts on the right summarize the total number of elements belonging to three functional categories: plant hormone response, abiotic/biotic stress response, and plant growth and development.

Promoter cis-element analysis predicts diverse regulatory functions for *GhCCR* genes. Analysis of the 2000 bp promoter regions using the PlantCARE database identified numerous cis-acting elements (Figure 7). These elements were classified into three functional categories: abiotic/biotic stress response, phytohormone response, and plant growth/development.

Notably, promoters of specific genes are enriched in elements associated with particular functions. For example, *GhCCR7* contains a high frequency of stress-responsive elements (e.g., MYB, MYC, ARE), suggesting a possible role in stress adaptation that requires experimental confirmation. In contrast, the promoter of *GhCCR64* is enriched with ABRE elements (involved in abscisic acid signaling) and multiple G-box elements, implying a potential link to ABA signaling; however, direct experimental evidence is needed to confirm functional regulation.

### 2.5. Transcriptome Analysis of the G. hirsutum GhCCR Gene Family

**Figure 8:**
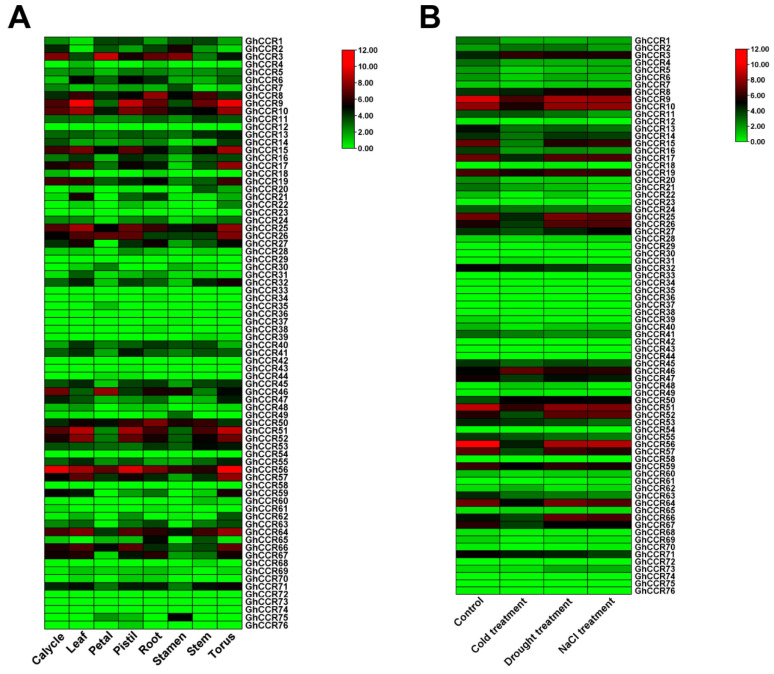
Expression profiles of *GhCCR* genes under various conditions: (**A**) Expression patterns across eight different tissues and developmental stages. (**B**) Expression patterns under three abiotic stress treatments: drought, cold, and salt. The color bars represent the scale of relative expression levels (log^2^-transformed FPKM or normalized values).

To elucidate the potential roles of *GhCCR* genes in development and stress adaptation, their expression profiles were analyzed using publicly available transcriptome data. Heatmaps were generated to visualize expression patterns across eight different cotton tissues and under three abiotic stress conditions: salinity, cold, and drought (Figure 8A,B).

The analysis revealed distinct expression patterns. In tissues, most *GhCCR* genes showed low expression (e.g., *GhCCR36/37*), while a few, such as *GhCCR10* and *GhCCR56*, were highly expressed in almost all plant tissues, suggesting tissue-specific functions in growth and development. Under abiotic stress, approximately half of the genes remained lowly expressed. However, several genes, including *GhCCR3* and *GhCCR50*, were significantly upregulated, indicating their potential involvement in stress response pathways.

### 2.6. Comparative Expression Analysis of CCR Genes Between Stiff-Stem (JY-25) and Soft-Stem (JR15) Cotton Lines

To investigate the association between *CCR* genes and stem mechanical strength in cotton, we performed RT-qPCR analysis of eight selected *GhCCR* candidates in two contrasting lines: the stiff-stem line *JY-25* and the soft-stem line *JR-15*. In the *JY-25* strain, the contents of lignin and hemicellulose were both higher than those in the *JR-15* strain, while the cellulose content showed no significant difference (Figure S1). The lignin content was directly quantified using the acetyl bromide method. The results revealed that *GhCCR25*, *GhCCR52* and *GhCCR64* exhibited higher expression levels in the stems of *JY-25* compared to *JR-15*. While *GhCCR8* showed elevated expression specifically in the roots of *JY-25*, the expression of *GhCCR45* did not differ significantly between the two lines. Meanwhile, the expression levels of *GhCCR3*, *GhCCR27*, and *GhCCR50* also showed little difference between the two cotton lines. Notably, *GhCCR25/52/64* expression was markedly upregulated in the stems of *JY-25*, suggesting a potential association with stem rigidity; however, functional validation is required to establish a causal role.

## 3. Discussion

Mechanistically, *CCR* catalyzes the reduction in cinnamoyl-CoA to cinnamaldehyde, the first committed step in the monolignol biosynthesis pathway. This reaction not only determines the total lignin content but also affects the ratio of guaiacyl (G) to syringyl (S) monomers, which profoundly influences cell wall mechanical properties, including stem bending strength and fracture resistance [13,18]. The number of *GhCCR* gene family members identified in this study (76) shows significant variation compared to species such as *A. thaliana* (11), *P. trichocarpa* (11), *O. sativa* (33), and *T. aestivum* (115). These numerical differences across species reflect key evolutionary events such as whole-genome duplication and subsequent gene loss within the *CCR* family [22,24]. On this basis, the member expansion of the *GhCCR* family is closely associated with the allopolyploidization history of *G. hirsutum*, which drives gene family expansion and leads to obvious interspecific numerical differences. Moreover, the expanded *GhCCR* genes have undergone functional divergence, providing abundant genetic resources for cotton to adapt to variable environmental stresses and maintain normal growth and development. Furthermore, *GhCCR* members exhibited an obvious uneven and non-random distribution across different cotton chromosomes. Such chromosomal distribution pattern is not a random event, but closely associated with genome evolution, segmental duplication events, and chromosomal rearrangement. The uneven enrichment of *GhCCR* genes on certain chromosomes implies that these chromosomes may serve as critical carriers for the expansion and functional differentiation of the *CCR* gene family, and further suggests that the clustered distribution of *GhCCR* members on partial chromosomes may be conducive to the coordinated expression and functional collaboration of adjacent genes involved in lignin biosynthesis and stress adaptation. Over time, homologous gene differentiation driven by segmental duplication (SD) and tandem duplication (TD) may have led to novel gene structures and functions [30]. As an allotetraploid, *G. hirsutum* harbors 58 segmentally duplicated gene pairs and 9 tandem repeat clusters, likely linked to its polyploidization history and evolutionary forces (Figure 1 and Figure 5). Sequence alignment analysis indicates that *GhCCR9* and *GhCCR51* in *G. hirsutum* share a closer evolutionary relationship with *AtCCR1* in *A. thaliana*. These genes also contain the highest number of conserved motifs and show closer phylogenetic relationships with *CCR* genes from most other crops (Figure 4A). These results indicate that Subfamily III, to which *GhCCR9/51* belong, may retain a conserved function associated with lignin biosynthesis. We also searched for *AtCCR2* homologs among *GhCCR* genes and identified *GhCCR8* and *GhCCR50* (Figure S2), which share high sequence and structural similarity with *AtCCR2*. These genes are upregulated under multiple abiotic stresses (Figure 8B), aligning with the reported stress-inducible role of *AtCCR2*. In contrast, our candidate genes *GhCCR25/52/64* are more closely related to *AtCCR1* (Figure 4A), supporting their potential role in constitutive lignification and stem strength. It is widely recognized that even genes within the same gene family frequently differ in their functions. Accordingly, we hypothesize that the other three *GhCCR* subfamilies in *G. hirsutum* may have undergone functional divergence during long-term evolution and thereby participate in diverse biological processes.

Plant *CCR* genes universally harbor a conserved NAD(P)H-binding domain (VTGALFGKT) and a characteristic structural motif, which are central not only to lignin biosynthesis but also to the cross-regulation between plant development and defense responses [11]. Within the *GhCCR* family, most genes clustered on the same phylogenetic branch share identical conserved motifs and exhibit highly similar gene structures, including consistent numbers of exons and introns (Figure 3D). The gene architecture characterized by fewer and shorter introns in the *GhCCR* family suggests a potential association with more rapid transcriptional responsiveness to environmental changes, which may confer enhanced functional efficiency under stress [31]. Comparative synteny analysis revealed that the *GhCCR* family exhibits the highest number of collinear gene pairs with *P. trichocarpa* (69), followed by *M. domestica* (55) and *T. cacao* (55). Among herbaceous dicots, *B. napus* (allotetraploid) shows 43 collinear pairs, while *C. sativus* and *M. sativa* have 41 and 29 pairs, respectively (Figure 6 and Figure S3). Collinearity counts with these herbaceous dicots are generally higher than those with the monocots *O. sativa* (14) and *Z. mays* (9), but remain lower than with *P. trichocarpa*. These findings indicate that the degree of synteny is influenced by multiple factors, including phylogenetic distance, genome duplication history (cotton, *B. napus*, and *M. sativa* are polyploids), and lineage-specific retention of duplicated genes. Thus, the higher collinearity with *P. trichocarpa* likely reflects both close taxonomic relatedness and shared genomic features, rather than a generic property of woody plants.

Promoters are key regulatory DNA sequences upstream of gene coding regions and central hubs in gene expression networks, making the analysis of their cis-acting elements fundamental for predicting gene function and deciphering regulatory logic [32,33]. This study shows that the promoters of the *GhCCR* gene family contain diverse cis-regulatory elements, which can be categorized into three groups: abiotic/biotic stress response, phytohormone response, and growth/development (Figure 7). Notably, the presence of ABRE and ERE elements in these promoters implies a possible link to abscisic acid (ABA) and ethylene signaling; however, cis-element prediction alone does not confirm functional regulation, and experimental validation is required to establish actual transcription factor binding and regulatory activity [31,34,35]. This aligns with known regulatory paradigms in other species: for example, *BnaA07.MYB43* in oilseed rape directly binds to the AC-II element in promoters of lignin biosynthesis genes like *BnaCCR1*, acting as a core transcriptional activator for vascular lignification; in rice, *OsCCR10* is directly activated by the transcription factor *OsNAC5* to enhance drought tolerance via lignin accumulation; and in flax, *LuCCR* family members are regulated by multiple hormones (ABA, MeJA, GA_3_, auxin), indicating coordinated hormonal control over developmental lignification and stress defense [25,36,37]. The functional importance of *CCR* genes is underscored by mutant phenotypes, such as the knockout of *AtCCR1* in *A. thaliana*, which leads to a 50% reduction in lignin, collapsed vessels, and dwarfism [29,38]. In our study, promoters of genes like *GhCCR16*, *GhCCR42*, *GhCCR64*, and *GhCCR74* contain abundant growth-related elements, suggesting these genes perform specific functions during cotton development. Furthermore, the enrichment of hormone response elements (e.g., ABRE) in the promoters of *GhCCR16*, *GhCCR42*, *GhCCR64* and *GhCCR76* implies that these specific *GhCCR* members may be involved in abiotic stress responses through ABA/JA signaling pathways. Integrating these observations, the enrichment of ABRE and MYB elements in *GhCCR25/52/64* promoters suggests a potential regulatory link between ABA signaling and lignin biosynthesis, which may enhance stem rigidity through stress-induced lignification [25,36].

**Figure 9:**
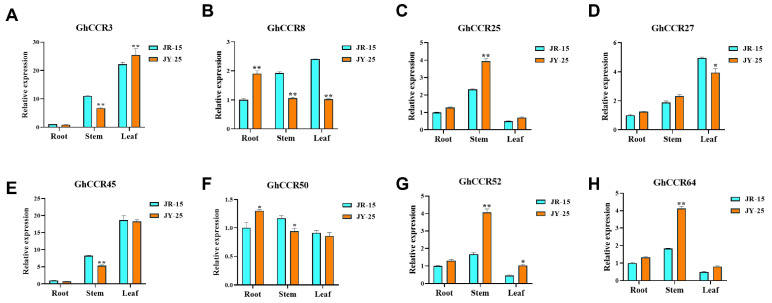
Relative expression levels of eight selected *GhCCR* genes in stiff-stem (*JY-25*) and soft-stem (*JR-15*) cotton lines. (**A**–**H**) Expression levels of *GhCCR3*, *GhCCR8*, *GhCCR25*, *GhCCR27*, *GhCCR45*, *GhCCR50*, *GhCCR52*, and *GhCCR64*, respectively. Data are presented as mean ± SD (n = 3). Asterisks indicate significant differences determined by one-way ANOVA followed by LSD post hoc test: * *p* < 0.05, ** *p* < 0.01.

RNA-seq has significantly advanced our understanding of gene family functions, evolution, and their role in biological adaptation by bridging genomic sequences with complex plant phenotypes [39]. *CCR* gene expression exhibits distinct tissue-specific patterns: in soybean, *GmCCR12* shows peak abundance in roots and high stem expression, while *GmCCR9* is primarily expressed in stems and developing seeds; in *A. thaliana*, *AtCCR1* displays low leaf expression but significantly higher levels in stems [11,40]. In *G. hirsutum*, *GhCCR9* is highly expressed in leaf, pistil, and torus tissues but lower in petals, whereas *GhCCR56* shows elevated expression in all cotton tissues. Notably, compared with other subfamilies, the *GhCCR* III subfamily exhibited significantly higher expression levels throughout the growth period, suggesting that its members act as key factors in cotton growth and development (Figure 8A). *CCR* genes are also crucial for abiotic stress responses, where enhanced lignin synthesis strengthens structural integrity. For example, *LuCCR2/5/10/18* are upregulated under salt, alkaline, and drought stress in flax [36]. Similarly, in cotton, genes such as *GhCCR3*, *GhCCR8* and *GhCCR50* show elevated expression across drought, cold, and salt stresses, indicating a broad role in abiotic stress resistance (Figure 8B). In contrast, *GhCCR46* is significantly upregulated only under cold stress, and *GhCCR66* responds to all three stresses—upregulated under drought and salt stress but downregulated under cold stress—highlighting their distinct functional specialization. Furthermore, RT-qPCR analysis revealed that *GhCCR25/GhCCR52/GhCCR64* exhibited higher expression in the roots, stems, and leaves of the hard-stemmed line *JY-25* compared with the soft-stemmed line *JR-15*; however, the difference reached a highly significant level only in the stems. The pronounced upregulation of *GhCCR25/GhCCR52/GhCCR64* in *JY-25* stems strongly suggests its potential key role in conferring stem rigidity (Figure 9). Therefore, *GhCCR25/GhCCR52/GhCCR64* emerges as prime candidates for future studies aimed at elucidating the molecular pathways that enhance stem strength in cotton. It should be emphasized that our findings are derived from transcriptomic and expression data only. Direct biochemical and genetic experiments (e.g., lignin quantification, histochemical staining, and transgenic assays) are required to confirm the functional involvement of the candidate *GhCCR* genes.

Collectively, our results establish a solid foundation for understanding the *GhCCR* gene family in cotton, highlighting *GhCCR25*, *GhCCR52*, and *GhCCR64* as valuable targets for breeding programs focused on improving stem mechanical strength. The conservation of stress-responsive cis-elements across plant species suggests that insights from model systems can inform cotton improvement. Future work should functionally validate these candidate genes via overexpression and knockout experiments, along with detailed phenotyping of lignin content, stem strength, and field-based agronomic traits. Such efforts will facilitate the development of cotton varieties with enhanced lodging resistance and stress tolerance, addressing critical challenges in modern cotton production.

## 4. Materials and Methods

### 4.1. Experimental Materials

In this study, two cotton lines: a stiff-stem line (*JY-25*) and a soft-stem line (*JR-15*) were used. Seeds were first soaked in water for 16–24 h and then sown in a soil mixture (peat moss: vermiculite: nutrient soil = 1:1:1). Plants were cultured under controlled conditions (25 ± 3 °C, 16 h light/8 h dark) for 4–6 weeks [41]. After this period, roots, stems, and leaves were collected for RNA extraction and subsequent RT-qPCR analysis.

### 4.2. Identification and Characterization of the GhCCR Gene Family in G. hirsutum

The reference genome and corresponding amino acid sequences of *G. hirsutum* TM-1 were retrieved from the COTTONOMICS database (https://cotton.zju.edu.cn/, accessed on 5 April 2025) [42,43]. Known *CCR* family protein sequences from the model species *Arabidopsis thaliana* were downloaded and used as query templates for a homology search against the *G. hirsutum* protein dataset to identify candidate *GhCCR* gene family members. Identified members were systematically named *GhCCR1* to *GhCCR76* based on their chromosomal positions. Gene location data and chromosome sizes were obtained from the COTTONOMICS database, and their physical positions were visualized using the TBtools Gene Location Visualize module (TBtools_windows-x64_2_376, South China Agricultural University, Guangzhou, China) [44,45]. The basic physicochemical properties of the predicted GhCCR proteins were analyzed using the DTU Health Tech online server (https://services.healthtech.dtu.dk/, accessed on 6 April 2025), while their subcellular localizations were predicted via the Plant-mPLoc website (http://www.csbio.sjtu.edu.cn/bioinf/plant-multi/, accessed on 6 May 2025) [46].

### 4.3. Sequence Alignment and Phylogenetic Analysis of the GhCCR Gene Family

A phylogenetic tree of the *GhCCR* gene family was constructed using MEGA7.0 (version 7.0.26) software based on the Neighbor-Joining (NJ) method. Branch support was evaluated through 5000 bootstrap replications [47]. The resulting Newick file was imported into the iTOL online platform (https://itol.embl.de/, accessed on 15 May 2025) for tree visualization. For comparative analysis, CCR protein sequences from closely related *Gossypium* species—*Gossypium tomentosum* (*GtCCR*, *Gotom.A05G181700*), *Gossypium mustelinum* (*GmCCR*, *Gomus.A05G179300*), and *Gossypium raimondii* (*GrCCR*, *Gorai.009G175500*)—were retrieved from the Phytozome13 database (https://phytozome-next.jgi.doe.gov/, accessed on 14 July 2025) [48]. Additionally, sequences from representative monocots, *Oryza sativa* (*OsCCR*, *LOC_Os04g53920*) and *Zea mays* (*ZmCCR*, *Zm00001d026370*) were included. Multiple sequence alignment was performed using ClustalW (https://www.genome.jp/tools-bin/clustalw, accessed on 15 July 2025), and the results were visualized with ENDscript/ESPrip (https://espript.ibcp.fr/ESPript/ESPript/index.php, accessed on 19 July 2025).

### 4.4. Analysis of Conserved Motifs, Conserved Domains and Gene Structures Within the GhCCR Gene Family

The GhCCR protein sequences were analyzed using MEME Suite (version 5.5.5) to identify conserved motifs, with the number of motifs set to 10 [49]. To investigate the structural characteristics of the *GhCCR* gene family, gene structure visualization was performed. The gff3 annotation file and a file containing all *GhCCR* gene IDs were loaded into TBtools to visualize exon–intron structures. Concurrently, the protein sequences were queried against the NCBI protein database to obtain hit data. Finally, these datasets were integrated using the TBtools Gene Structure View (Advanced) function to generate a combined phylogenetic tree, conserved motif map, and gene structure schematic [50].

### 4.5. Collinearity Analysis of the GhCCR Gene Family

We performed genome-wide collinearity analysis for the study species (*G. hirsutum*) using the “One Step MCScanX” function in TBtools with default parameters. Default parameters were applied: match score 50, gap penalty^−1^, and an E-value cutoff of 1 × 10^−5^. To investigate interspecies homology, we further conducted comparative genomic analyses between *G. hirsutum* and several other plant species. The analyses involved processing the corresponding genomic FASTA and GFF3 annotation files, which generated three core output files: a GFF file, a CTL file, and a Collinearity file. Prior to visualization, chloroplast and mitochondrial sequences were manually identified and removed from the CTL file, and the file was reordered accordingly. All results were finally visualized using the plotting functions within TBtools [33].

### 4.6. Analysis of Promoter Cis-Acting Elements in the GhCCR Gene Family

Cis-acting regulatory elements in the 2000 bp promoter region upstream of each *GhCCR* gene were predicted using the PlantCARE database (version 1.0, accessed on 20 March 2025) online database. The occurrence count of each element type was quantified, and the results were visualized as a heatmap using TBtools [51].

### 4.7. Transcriptome Analysis of the GhCCR Gene Family

The transcriptome dataset for the *GhCCR* gene family was obtained from the publicly available COTTONOMICS database (http://cotton.zju.edu.cn/, accessed on 22 July 2025). The raw RNA-seq data have been deposited in the NCBI Sequence Read Archive (SRA) under accession number PRJNA248163. Although the original dataset lacked technical replicates for stress conditions, three biological replicates were included per treatment. This dataset includes FPKM expression values across eight distinct tissues (e.g., leaf, root, stem) and under three abiotic stress conditions (PEG-simulated drought, salt, and cold stress) in *G. hirsutum* (Table S4). An expression heatmap was generated using the HeatMap function in TBtools to visualize expression patterns. Row-scale normalization was applied with default settings for all other parameters [52].

### 4.8. Quantitative Real-Time Polymerase Chain Reaction (RT-qPCR) Analysis of the GhCCR Gene Family

RNA extraction was performed using the Nuoto^®^ AutoExtracter-32 Nucleic Acid Extractor with the 5 fz PCR DNA/RNA AutoPurification Kit from Kangma-Healthcode (Kangma-Healthcode Biotech Co., Ltd., Shanghai, China), and subsequently reverse transcribed into first-strand cDNA using the AT311 kit (TransScript^®^ One-Step gDNA Removal and cDNA Synthesis SuperMix (AT311), TransGen Biotech Co., Ltd., Beijing, China). Specific primers were designed with NCBI Primer-BLAST (Table S5). RT-qPCR was performed using 2× SuperFast Universal SYBR Master Mix (TransGen Biotech Co., Ltd., Beijing, China) on a Bio-Rad CFX96 Touch™ (Bio-Rad Laboratories, Hercules, CA, USA) system under the following conditions: initial denaturation at 95 °C for 15 min, followed by 40 cycles of 95 °C for 10 s, 60 °C for 30 s, and 72 °C for 30 s [53]. Relative expression levels were calculated via the 2^−∆∆CT^ method, normalizing to the internal reference gene *GhUBQ7* (*Ghir_D12G021700*) [41]. Three independent biological replicates were performed, each consisting of pooled root, stem, and leaf tissues collected from three individual plants. For each biological replicate, three technical replicates were run on the qPCR instrument to ensure measurement accuracy.

### 4.9. Statistical Analysis

RT-qPCR data analysis was performed using GraphPad Prism version 9.5.0 (GraphPad Software, Boston, MA, USA). Relative gene expression levels were evaluated through one-way analysis of variance (ANOVA), followed by LSD post hoc tests for multiple comparisons, with statistical significance set at * *p* < 0.05, ** *p* < 0.01. Prior to ANOVA, all data were assessed for normality and homogeneity of variance to ensure the validity of the statistical model [54].

## 5. Conclusions

This study identified and characterized 76 *GhCCR* family members in *G. hirsutum* through genome-wide analysis. These genes were classified into four subfamilies, and the genes within each subfamily exhibit highly conserved gene structures and evolutionary relationships. The expansion of the *GhCCR* family was driven by nine tandem duplication clusters and 58 segmental duplication events. Comparative genomic analysis revealed stronger collinearity and higher homology with woody plant species, highlighting potential evolutionary conservation. Cis-acting element analysis of promoter regions indicated that *GhCCR* genes are enriched with regulatory elements responsive to abiotic stress and plant growth/development, suggesting their involvement in these biological processes. Transcriptome profiling showed generally low expression of *GhCCR* genes across eight tissues and under three abiotic stress conditions. However, specific members exhibited distinct expression patterns: *GhCCR10* and *GhCCR56* showed high expression in almost all plant tissues, while *GhCCR3*, *GhCCR50* were upregulated under abiotic stress. RT-qPCR validation of eight selected genes in contrasting stem-hardness lines (stiff-stem *JY-25* vs. soft-stem *JR-15*) revealed that *GhCCR25/GhCCR52/GhCCR64* had significantly higher expression in the stem of *JY-25*. These three genes may be associated with lignin biosynthesis and stem strength modulation, but direct functional assays (e.g., transgenic studies) are needed to confirm their roles. Collectively, these findings provide a comprehensive genomic and transcriptional foundation for elucidating the biological functions of *GhCCR* genes in cotton development and abiotic stress response, with significant implications for improving lodging resistance through molecular breeding.

## Data Availability

All data supporting the conclusions of this paper are provided in the article and Supplementary Materials. Genome sequence data for all species are available in the Phytozome database (https://phytozome-next.jgi.doe.gov/) and CottonFGD database (https://cottonfgd.net/). Publicly available RNA-seq data are available on CottonFGD database.

## Supplementary Materials

The following supporting information can be downloaded at: https://www.mdpi.com/article/10.3390/life16050861/s1, Figure S1: Biochemical indices; Figure S2: Multiple sequence alignment and structural analysis of *GhCCR8* and *GhCCR50*; Figure S3: Comparative synteny analysis of *CCR* genes between *G. hirsutum* and three representative plant species. Table S1: List of *Gossypium_hirsutum GhGRF* genes; Table S2: Nine tandem repeat clusters; Table S3: Sequence and SeqLogo of the Motif1-10; Table S4: Expression of *GhCCR* genes; Table S5: Primers used for RT-qPCR in the article.

## Abbreviations

The following abbreviations are used in this manuscript:

CCR	Cinnamoyl-CoA Reducase
SD	Segmental Duplication
TD	Tandem Duplication
ABA	Abscisic Acid
ROS	Reactive Oxygen Species
PAL	Phenylalanine Ammonia-Lyase
CAD	Cinnamyl Alcohol Dehydrogenase
MeJA	Methyl Jasmonate
GA_3_	Gibberellin A3

## References

[1] Li H., Zhang S., Zhao Y., Zhao X., Xie W., Guo Y., Wang Y., Li K., Guo J., Zhu Q.-H. (2022). Identification and Characterization of Cinnamyl Alcohol Dehydrogenase Encoding Genes Involved in Lignin Biosynthesis and Resistance to *Verticillium dahliae* in Upland Cotton (*Gossypium hirsutum* L.). Front. Plant Sci..

[2] Wen L., Li W., Parris S., West M., Lawson J., Smathers M., Li Z., Jones D., Jin S., Saski C.A. (2020). Transcriptomic profiles of non-embryogenic and embryogenic callus cells in a highly regenerative upland cotton line (*Gossypium hirsutum* L.). BMC Dev. Biol..

[3] Bano N., Fakhrah S., Mohanty C.S., Bag S.K. (2022). Transcriptome Meta-Analysis Associated Targeting *Hub* Genes and Pathways of Drought and Salt Stress Responses in Cotton (*Gossypium hirsutum*): A Network Biology Approach. Front. Plant Sci..

[4] Feng S., Long X., Gao M., Zhao Y., Guan X. (2023). Global identification of natural antisense transcripts in *Gossypium hirsutum* and *Gossypium barbadense* under chilling stress. iScience.

[5] Guo C., Zhu L., Sun H., Han Q., Wang S., Zhu J., Zhang Y., Zhang K., Bai Z., Li A. (2024). Evaluation of drought-tolerant varieties based on root system architecture in cotton (*Gossypium hirsutum* L.). BMC Plant Biol..

[6] Han Y., Yang Y., Luo H., Cui J., Kuang B., Zhang X., Sun J., Xu J.W., Liu F. (2025). Water stress reduces cellulose deposition in the cell wall and increases wax content, resulting in decreased fiber quality. Front. Plant Sci..

[7] Jin X., Chai Q., Liu C., Niu X., Li W., Shang X., Gu A., Zhang D., Guo W. (2023). Cotton *GhNAC4* promotes drought tolerance by regulating secondary cell wall biosynthesis and ribosomal protein homeostasis. Plant J..

[8] Lai Z., Zhang K., Liao Z., Kou H., Pei S., Dou Z., Bai Z., Fan J. (2023). Stem Hydraulic Conductance, Leaf Photosynthesis, and Carbon Metabolism Responses of Cotton to Short-Term Drought and Rewatering. Agronomy.

[9] Wang D.R., Venturas M.D., Mackay D.S., Hunsaker D.J., Thorp K.R., Gore M.A., Pauli D. (2020). Use of hydraulic traits for modeling genotype-specific acclimation in cotton under drought. New Phytol..

[10] Guo A.-H., Su Y., Huang Y., Wang Y.-M., Nie H.-S., Zhao N., Hua J.-P. (2021). QTL controlling fiber quality traits under salt stress in upland cotton (*Gossypium hirsutum* L.). Theor. Appl. Genet..

[11] De Meester B., de Vries L., Özparpucu M., Gierlinger N., Corneillie S., Pallidis A., Goeminne G., Morreel K., De Bruyne M., De Rycke R. (2018). Vessel-Specific Reintroduction of CINNAMOYL-COA REDUCTASE1 (CCR1) in Dwarfed *ccr1* Mutants Restores Vessel and Xylary Fiber Integrity and Increases Biomass. Plant Physiol..

[12] Bellincampi D., Cervone F., Lionetti V. (2014). Plant cell wall dynamics and wall-related susceptibility in plant-pathogen interactions. Front. Plant Sci..

[13] Liu Q.Q., Luo L., Zheng L.Q. (2018). Lignins: Biosynthesis and Biological Functions in Plants. Int. J. Mol. Sci..

[14] Oelmüller R., Tseng Y.-H., Gandhi A. (2023). Signals and Their Perception for Remodelling, Adjustment and Repair of the Plant Cell Wall. Int. J. Mol. Sci..

[15] Wolf S., Hématy K., Höfte H. (2012). Growth Control and Cell Wall Signaling in Plants. Annu. Rev. Plant Biol..

[16] Amjad M., Wang Y., Han S., Haider M.Z., Sami A., Batool A., Shafiq M., Ali Q., Dong J., Sabir I.A. (2024). Genome wide identification of *phenylalanine ammonia-lyase* (*PAL*) gene family in *Cucumis sativus* (cucumber) against abiotic stress. BMC Genom. Data.

[17] Liu S., Zhao L., Liao Y., Luo Z., Wang H., Wang P., Zhao H., Xia J., Huang C.F. (2020). Dysfunction of the 4-coumarate:coenzyme A ligase *4CL4* impacts aluminum resistance and lignin accumulation in rice. Plant J..

[18] Chao N., Li N., Qi Q., Li S., Lv T., Jiang X.-N., Gai Y. (2016). Characterization of the *cinnamoyl-CoA reductase* (*CCR*) gene family in *Populus tomentosa* reveals the enzymatic active sites and evolution of *CCR*. Planta.

[19] An F., Chen T., Zhu W., Xiao X., Xue J., Luo X., Wei Z., Li K., Chen S., Cai J. (2024). Systematic Analysis of Cinnamyl Alcohol Dehydrogenase Family in Cassava and Validation of *MeCAD13* and *MeCAD28* in Lignin Synthesis and Postharvest Physiological Deterioration. Int. J. Mol. Sci..

[20] Rong W., Luo M., Shan T., Wei X., Du L., Xu H., Zhang Z. (2016). A Wheat Cinnamyl Alcohol Dehydrogenase *TaCAD12* Contributes to Host Resistance to the Sharp Eyespot Disease. Front. Plant Sci..

[21] Mo F., Li L., Zhang C., Yang C., Chen G., Niu Y., Si J., Liu T., Sun X., Wang S. (2022). Genome-Wide Analysis and Expression Profiling of the *Phenylalanine Ammonia-Lyase* Gene Family in *Solanum tuberosum*. Int. J. Mol. Sci..

[22] Zhan W., Cui L., Song N., Liu X., Guo G., Zhang Y. (2025). Comprehensive analysis of *cinnamoyl-CoA reductase* (*CCR*) gene family in wheat: Implications for lignin biosynthesis and stress responses. BMC Plant Biol..

[23] Wu D., Ni M., Lei X., Zhang L., Zhang W., Shu H., Wang Z., Zhu J., Cheng S., Liu P. (2022). Analyses of Pepper *Cinnamoyl-CoA Reductase* Gene Family and Cloning of *CcCCR1/2* and Their Function Identification in the Formation of Pungency. Horticulturae.

[24] Wang Y., Xu J., Zhao W., Li J., Chen J. (2022). Genome-wide identification, characterization, and genetic diversity of *CCR* gene family in *Dalbergia odorifera*. Front. Plant Sci..

[25] Bang S.W., Choi S., Jin X., Jung S.E., Choi J.W., Seo J.S., Kim J.K. (2021). Transcriptional activation of rice CINNAMOYL-CoA REDUCTASE 10 by *OsNAC5*, contributes to drought tolerance by modulating lignin accumulation in roots. Plant Biotechnol. J..

[26] Park H.L., Bhoo S.H., Kwon M., Lee S.-W., Cho M.-H. (2017). Biochemical and Expression Analyses of the Rice *Cinnamoyl-CoA Reductase* Gene Family. Front. Plant Sci..

[27] Zhang Y., Zhang H., Zhao H., Xia Y., Zheng X., Fan R., Tan Z., Duan C., Fu Y., Li L. (2022). Multi-omics analysis dissects the genetic architecture of seed coat content in *Brassica napus*. Genome Biol..

[28] Huang S., Kang X., Fu R., Zheng L., Li P., Tang F., Chao N., Liu L. (2024). Simultaneous Down-Regulation of Dominant Cinnamoyl CoA Reductase and Cinnamyl Alcohol Dehydrogenase Dramatically Altered Lignin Content in Mulberry. Plants.

[29] Tamasloukht B., Wong Quai Lam M.S.-J., Martinez Y., Tozo K., Barbier O., Jourda C., Jauneau A., Borderies G., Balzergue S., Renou J.-P. (2011). Characterization of a cinnamoyl-CoA reductase 1 (CCR1) mutant in maize: Effects on lignification, fibre development, and global gene expression. J. Exp. Bot..

[30] Cannon S.B., Mitra A., Baumgarten A., Young N.D., May G. (2004). The roles of segmental and tandem gene duplication in the evolution of large gene families in *Arabidopsis thaliana*. BMC Plant Biol..

[31] Ferrelli M.L., Pidre M.L., Ghiringhelli P.D., Torres S., Fabre M.L., Masson T., Cédola M.T., Sciocco-Cap A., Romanowski V. (2018). Genomic analysis of an Argentinean isolate of *Spodoptera frugiperda* granulovirus reveals that various baculoviruses code for Lef-7 proteins with three F-box domains. PLoS ONE.

[32] Tang F., Tu H., Shang Q., Gao X., Liang P. (2020). Molecular Cloning and Characterization of Five *Glutathione S-Transferase* Genes and Promoters from *Micromelalopha troglodyta* (Graeser) (Lepidoptera: Notodontidae) and Their Response to Tannic Acid Stress. Insects.

[33] He F., Shi Y.-J., Li J.-L., Lin T.-T., Zhao K.-J., Chen L.-H., Mi J.-X., Zhang F., Zhong Y., Lu M.-M. (2022). Genome-wide analysis and expression profiling of Cation/H^+^ exchanger (CAX) family genes reveal likely functions in cadmium stress responses in poplar. Int. J. Biol. Macromol..

[34] Lankinen Å., Abreha K.B., Alexandersson E., Andersson S., Andreasson E. (2016). Nongenetic Inheritance of Induced Resistance in a Wild Annual Plant. Phytopathology.

[35] Chan Z., Cao P.B., Azar S., SanClemente H., Mounet F., Dunand C., Marque G., Marque C., Teulières C. (2015). Genome-Wide Analysis of the AP2/ERF Family in *Eucalyptus grandis*: An Intriguing Over-Representation of Stress-Responsive *DREB1/CBF* Genes. PLoS ONE.

[36] Song X., Liu D., Yao Y., Tang L., Cheng L., Yang L., Jiang Z., Kang Q., Chen S., Ru J. (2025). Genome-wide identification and expression pattern analysis of the *cinnamoyl-CoA reductase* gene family in flax (*Linum usitatissimum* L.). BMC Genom..

[37] Stein A., Coriton O., Rousseau-Gueutin M., Samans B., Schiessl S.V., Obermeier C., Parkin I.A.P., Chèvre A.M., Snowdon R.J. (2017). Mapping of homoeologous chromosome exchanges influencing quantitative trait variation in *Brassica napus*. Plant Biotechnol. J..

[38] De Meester B., Vanholme R., de Vries L., Wouters M., Van Doorsselaere J., Boerjan W. (2021). Vessel- and ray-specific monolignol biosynthesis as an approach to engineer fiber-hypolignification and enhanced saccharification in poplar. Plant J..

[39] Hosseini S., Ramezanpour S., Soltanloo H., Seifati E. (2023). RNA-seq analysis and reconstruction of gene networks involved in response to salinity stress in quinoa (*cv.* Titicaca). Sci. Rep..

[40] Li X., Li Y., Li S., Sun M., Cai Q., Sun Y., Li S., Yin Y., Yu T., Zhang J. (2025). Genome-wide characterization and stress-responsive expression analysis of the cinnamoyl-CoA reductase gene family in soybean. Front. Plant Sci..

[41] Zhang S., Cai X., Wei J., Wang H., Liu C., Li X., Tang L., Zhou X., Zhang J. (2024). GhWRKY40 interacts with an asparaginase GhAP_D6_ involved in fiber development in upland cotton (*Gossypium hirsutum* L.). Genes.

[42] Dai F., Chen J., Zhang Z., Liu F., Li J., Zhao T., Hu Y., Zhang T., Fang L. (2022). COTTONOMICS: A comprehensive cotton multi-omics database. Database.

[43] Zhang Z., Chai M., Yang Z., Yang Z., Fan L. (2022). GRAND: An Integrated Genome, Transcriptome Resources, and Gene Network Database for *Gossypium*. Front. Plant Sci..

[44] Chen C., Chen H., Zhang Y., Thomas H.R., Frank M.H., He Y., Xia R. (2020). TBtools: An Integrative Toolkit Developed for Interactive Analyses of Big Biological Data. Mol. Plant.

[45] Chen C., Wu Y., Li J., Wang X., Zeng Z., Xu J., Liu Y., Feng J., Chen H., He Y. (2023). TBtools-II: A “one for all, all for one” bioinformatics platform for biological big-data mining. Mol. Plant.

[46] Chou K., Shen H. (2010). Plant-mPLoc: A top-down strategy to augment the power for predicting plant protein subcellular localization. PLoS ONE.

[47] Hall B.G. (2013). Building Phylogenetic Trees from Molecular Data with MEGA. Mol. Biol. Evol..

[48] Goodstein D.M., Shu S., Howson R., Neupane R., Hayes R.D., Fazo J., Mitros T., Dirks W., Hellsten U., Putnam N. (2012). Phytozome: A comparative platform for green plant genomics. Nucleic Acids Res..

[49] Bailey T.L., Boden M., Buske F.A., Frith M., Grant C.E., Clementi L., Ren J., Li W.W., Noble W.S. (2009). MEME SUITE: Tools for motif discovery and searching. Nucleic Acids Res..

[50] Bao Y., Zhao R., Hu S., Li X., Wang L., Wang J., Ji J., Wang W., Zhu C., Chen J. (2025). Genome-Wide Identification and Expression Analysis of CrRLK1-like Gene Family in Potatoes (*Solanum tuberosum* L.) and Its Role in PAMP-Triggered Immunity. Genes.

[51] Gong Z., Wu X., Luo Y., Zhou T., Yang Z., Wu Y. (2025). Genome-Wide Identification and Analysis of the *JAZ* Gene Family in *Artemisia argyi*. Curr. Issues Mol. Biol..

[52] Bai J.-F., Wang Y.-K., Wang P., Yuan S.-H., Gao J.-G., Duan W.-J., Wang N., Zhang F.-T., Zhang W.-J., Qin M.-Y. (2018). Genome-wide identification and analysis of the *COI* gene family in wheat (*Triticum aestivum* L.). BMC Genom..

[53] Jiao Z., Wang J., Shi Y., Wang Z., Zhang J., Du Q., Liu B., Jia X., Niu J., Gu C. (2023). Genome-wide identification and analysis of the *EPF* gene family in *Sorghum bicolor* (L.) moench. Plants.

[54] Han S., Jiao Z., Niu M.-X., Yu X., Huang M., Liu C., Wang H.-L., Zhou Y., Mao W., Wang X. (2021). Genome-Wide Comprehensive Analysis of the *GASA* Gene Family in *Populus*. Int. J. Mol. Sci..

