# Supplementary material for: Genome-Wide Characterization and Expression Profiling of the CCR Gene Family Associated with Stem Strength in Upland Cotton (Gossypium hirsutum L.)
Source: Life (Basel). 2026 May 21;16(5):861. doi: 10.3390/life16050861 (PMC13208141; doi:10.3390/life16050861)
Supplement: Supplementary file 1 [file life-16-00861-s001.zip › Supplementary Diagram.pdf]

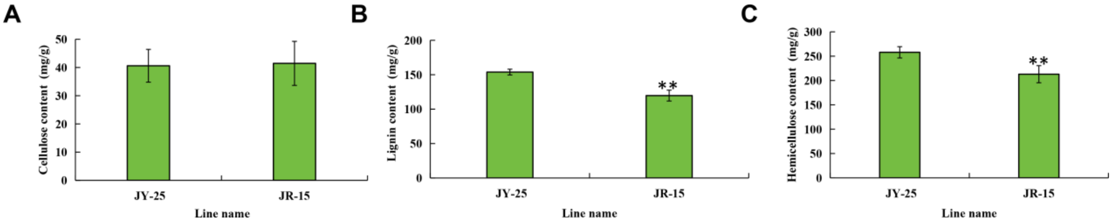

Figure S1. Physiological indicators of cotton. (A) Cellulose content; (B) Lignin content; (C) Hemicellulose content. Data were analyzed by one-way ANOVA with LSD post hoc test (n = 6). Asterisks indicate significant differences: *$p < 0.05$; **$p < 0.01$.

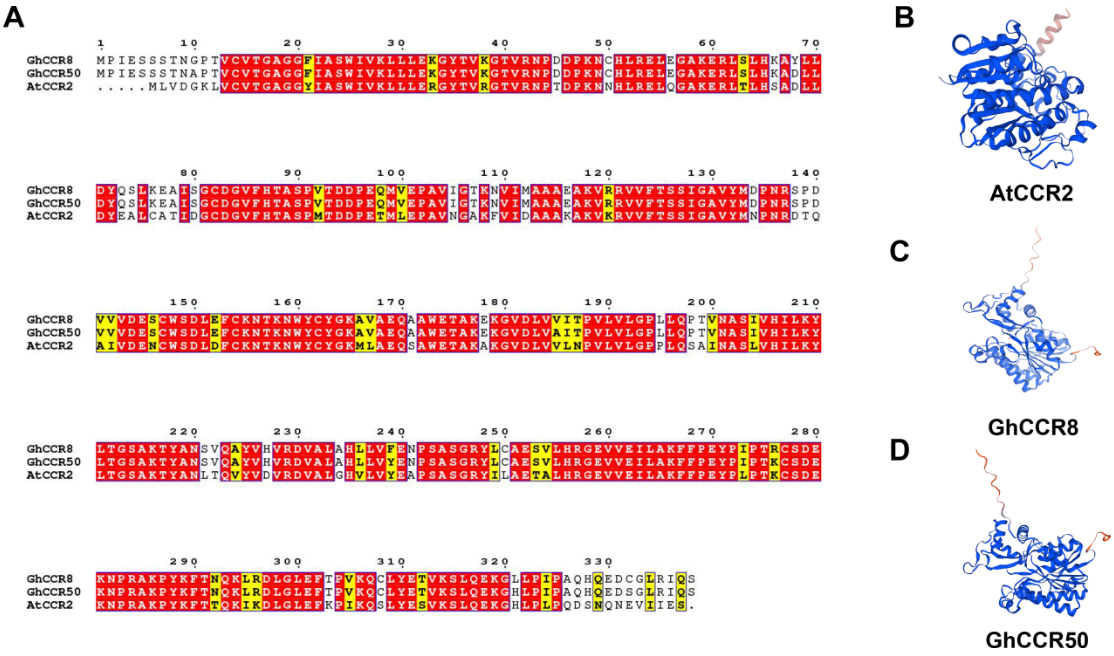

Figure S2. Multiple sequence alignment and structural analysis of *GhCCR8* and *GhCCR50*. (A) Red text denotes conserved sequences, with red highlighting indicating identical sequences. (B-D) Analysis of the tertiary structure of proteins encoded by three genes. (B) *AtCCR2*; (C) *GhCCR8*; (D) *GhCCR50*.

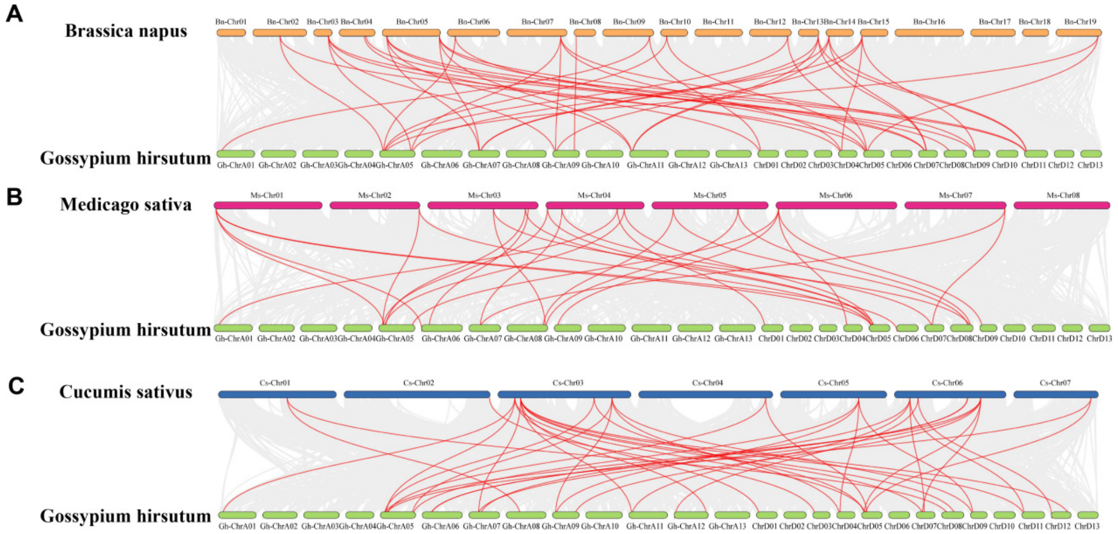

Figure S3. Comparative synteny analysis of *CCR* genes between *G. hirsutum* and three representative plant species. The grey lines in each panel represent systemic blocks between *G. hirsutum* and the compared species (*Brassica napus*, *Cucumis sativus* and *Medicago sativa*), while the red lines highlight homologous *CCR* gene pairs.
